# Cerebral venous sinus thrombosis after COVID-19 vaccination: a case report and literature review

**DOI:** 10.1093/omcr/omac154

**Published:** 2023-01-18

**Authors:** Mohamed Elfil, Mohammad Aladawi, Dmitry Balian, Ismail Fahad, Daniel J Zhou, Brian Villafuerte-Trisolini, Thomas Scott Diesing

**Affiliations:** Department of Neurological Sciences, University of Nebraska Medical Center, Omaha, NE, USA; Department of Neurological Sciences, University of Nebraska Medical Center, Omaha, NE, USA; Department of Neurological Sciences, University of Nebraska Medical Center, Omaha, NE, USA; Department of Neurological Sciences, University of Nebraska Medical Center, Omaha, NE, USA; Department of Neurological Sciences, University of Nebraska Medical Center, Omaha, NE, USA; Department of Neurological Sciences, University of Nebraska Medical Center, Omaha, NE, USA; Department of Neurological Sciences, University of Nebraska Medical Center, Omaha, NE, USA

## Abstract

As COVID-19 vaccines became widely available, there have been reports of neurovascular complications. In this article, we aim to report a case of cerebral venous sinus thrombosis (CVST) induced by COVID-19 vaccination, with a literature review on similar cases as well as the potential pathophysiological mechanisms. Our case is a healthy male who developed headache, vomiting, photophobia and diplopia after receiving the Ad26.COV2.S vaccine. Fundus examination showed papilledema, and magnetic resonance imaging of the brain and cerebral veins showed CVST involving the superior sagittal sinus and right transverse sinus extending into the right jugular vein. Hypercoagulability workup was unremarkable, and the patient received immunotherapy and anticoagulation. Following this treatment, symptoms resolved, and he had no residual neurologic deficits. Developing neurologic manifestations, especially severe headaches with papilledema, after COVID-19 vaccination should warrant neuroimaging. Early recognition and management of CVST are essential for good clinical outcomes.

## INTRODUCTION

Several vaccines for severe acute respiratory syndrome coronavirus 2 (SARS-CoV-2) became available for use during the COVID-19 pandemic. The Ad26.COV2.S vaccine is a recombinant replication-incompetent human adenovirus type 26 vector encoding full-length SARS-CoV-2 spike protein in a prefusion-stabilized conformation [1]. The ChAdOx1 nCoV-19 vaccine is another adenovirus-based vaccine that consists of a replication-deficient chimpanzee adenoviral vector ChAdOx1, containing the SARS-CoV-2 structural surface glycoprotein antigen (spike protein; nCoV-19) gene [2]. Both vaccines have been shown to be safe and efficacious in protecting against COVID-19 infection and reducing the risk of critical illness [3].

The most common side effects of COVID-19 vaccination include fatigue, headache and local pain around the injection site [4]. However, there have been rare cases of cerebral venous sinus thrombosis (CVST) associated with either the Ad26.COV2.S vaccine or ChAdOx1 nCoV-19 vaccine [5, 6]. CVST can present with headache or seizures and may involve elevated intracranial pressure [7]. When CVST follows a COVID-19 vaccination, it may be referred to as vaccine-induced immune thrombotic thrombocytopenia (VITT) [8]. The proposed mechanism of VITT is similar to that of heparin-induced thrombotic thrombocytopenia (HITT) in terms of developing high levels of antibodies against the complexes of platelet factor 4 (PF4) and heparin with associated thrombocytopenia [6]. However, the immune response is triggered by the vaccine and considered to be heparin independent [8]. Medical literature discussed many VITT cases to date. Our report also offers a VITT–related CVST case in a young man with no prior history of thrombosis. We also provide a literature review on CVST associated with adenovirus vector-based vaccines, with discussion of proposed mechanisms and recent treatment guidelines.

## CASE REPORT

A 28-year-old previously healthy man presented to the emergency room with severe bifrontal headaches for 2 days. The headaches were acute, throbbing and associated with blurred vision, diplopia, photophobia, nausea and vomiting, which worsened with coughing, bending forward and straining. His headaches were refractory to over-the-counter medications. He had no prior headaches. His maternal grandmother had a brain aneurysm diagnosed at the age of 50 years. He otherwise had no family history of headaches or clotting disorders. He was not on any medications but received the Ad26.COV2.S vaccine 10 days prior to symptom onset. A fundus exam revealed papilledema, and the rest of his physical and neurologic examination was otherwise unremarkable.

**Figure 1:**
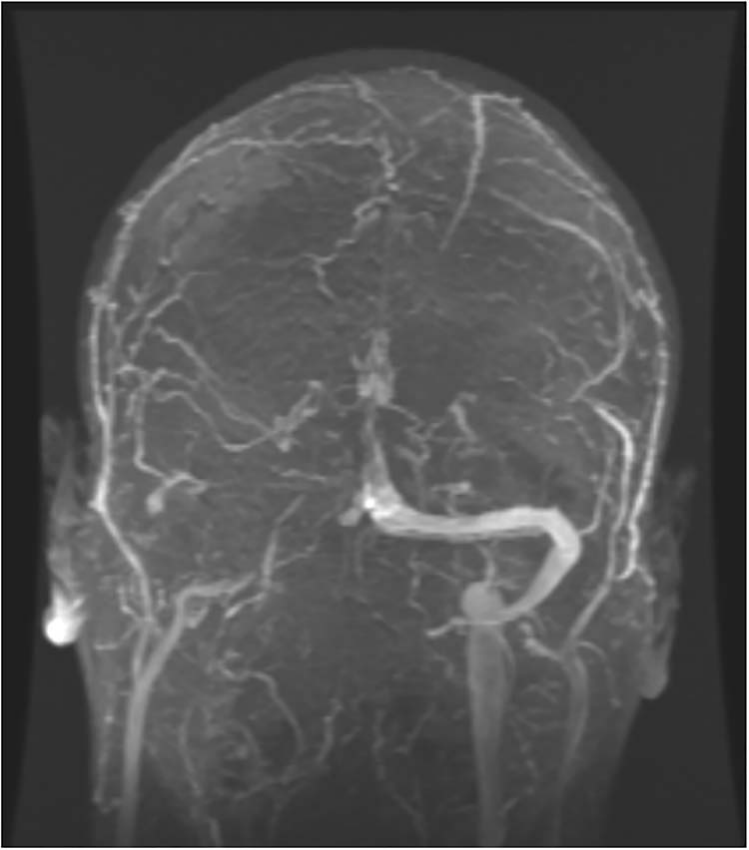
MRV filling defect within the superior sagittal sinus, as well as the right transverse sinus extending into the right jugular vein.

**Figure 2:**
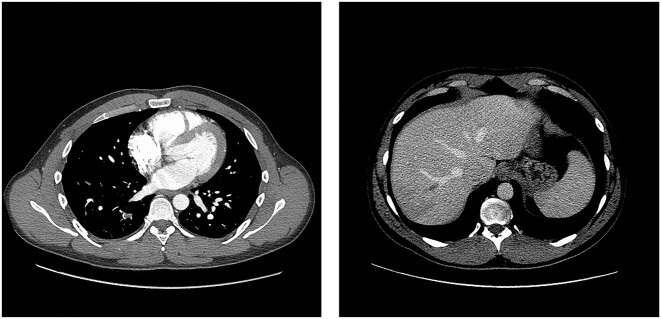
Shows multiple segmental and subsegmental pulmonary emboli throughout the right lung and occluded right hepatic vein with wedge-shaped hepatic segment hypodensity.

Magnetic resonance venography (MRV) of the brain showed filling defects within the superior sagittal sinus as well as the right transverse sinus extending into the right jugular vein (Fig. 1), thereby giving him the diagnosis of CVST. The initial laboratory workup was remarkable for platelet count 63 000/μL, international normalized ratio (INR) 1.5, partial thromboplastin time (PTT) 36.3 s and D-dimer 22 546 ng/mL. Computed tomography angiography (CTA) of the chest and abdomen showed multiple segmental and subsegmental pulmonary emboli throughout the right lung and occluded right hepatic vein with wedge-shaped hepatic segment hypodensity (Fig. 2). Venous duplex ultrasound of the bilateral lower extremities was unremarkable. An extensive workup for hypercoagulability was performed. Serum SARS-CoV-2 and PF4 antibodies were checked because of his recent COVID-19 vaccination and were positive. Along with ruling out other causes of hypercoagulability, patient's labs confirmed the suspicion for VITT. Noted that the routine COVID-19 PCR test for admission was negative.

Per the VITT protocol [9], the patient received intravenous immunoglobulins (IVIG) (1 g/kg/day) for 2 days and a continuous infusion of argatroban for 6 days, followed by oral dabigatran 150 mg two times daily. His headaches and nausea improved with supportive measures, and his diplopia soon resolved. He was discharged 2 days after being switched to oral anticoagulation. He did not have any residual neurologic deficits at the time of discharge, and normalization of platelet count was achieved prior to discharge.

## DISCUSSION

CVST has to date been reported in a small number of cases after receiving the SARS-CoV2 adenoviral vector vaccines (Table 1). In these cases, the typical onset of symptoms was 1–2 weeks after vaccination. The reported clinical features included new-onset severe persistent headache, focal neurologic symptoms, visual changes and seizures [10]. In most of the cases, other clinical findings including severe abdominal pain, fever and shortness of breath have accompanied the neurological symptoms, which was indicative of systemic thrombosis [11].

**Table 1:** Demographics, clinical presentation, treatment and outcomes in patients with CVST post-vector-based SARS-CoV2 vaccines administration

Author	Age and gender	Presenting symptoms	Duration between vaccination and symptom onset	Administered vaccine	Lowest platelet count (10^9^/L)	Clotting screen on admission	Peak D-Dimer levels	PF4 antibody ELISA	Neuroimaging findings	Extracranial thrombosis	Treatment	Residual neurologic deficits
Our case report	28 M	Headache Blurry vision Diplopia Photophobia	10 days	Ad26.COV2.S	63	PT: 18.3 INR: 1.5 APTT: 36.3	22 546	Positive	Thrombosis of superior sagittal and right transverse sinuses, and right IJV	Right segmental and subsegmental PE, Right hepatic vein thrombosis	Argatroban, dabigatran, and IVIG	Recovery with no residual neurologic deficits
Graf	29 M	Headache Aphasia Apraxia	9 days	ChAdOx1 nCoV-19	32	NA	65.7	NA	Left transverse, sigmoid sinuses and IJV thrombosis, Temporo-parietal ICH	Mesenteric and portal vein thrombosis	Argatroban and IVIG	Aphasia improved
Soleimani	34 M	Headache Photophobia Hemiparesis	13 days	ChAdOx1 nCoV-19	23	PT: 14.8 INR: 1.5 APTT: 23.9	37 293	Positive	Superior sagittal, bilateral transverse sinuses, and left vein of Trolard thrombosis, Right sided midline shit and uncal herniation	Right lower lobe segmental PE	PLEX, IVIG, argatroban, decompressive hemicraniectomy	Right hemiparesis and aphasia
Soleimani	59 F	Headache Hemiparesis Sensory loss Neglect Seizure	14 days	ChAdOx1 nCoV-19	18	PT: 14.5 INR: 1.2 APTT: 24.4	38 588	Positive	Superior sagittal and right transverse sinus thrombosis, Frontal hematoma and midline shift.	Thrombosed right hepatic vein and right lower lobe pulmonary emboli	IVIG, IVSM, argatroban, and decompressive hemicraniectomy	Left hemiparesis
Soleimani	39 F	Headache Photophobia	10 days	ChAdOx1 nCoV-19	63	PT: 15.1 INR: 1.1 APTT: 33.2	20 289	Negative	Left transverse, sigmoid, and sagittal sinuses and jugular venous thrombosis	None	IVSM, PLEX and argatroban	None
Zanferrari	40 F	Headache Aphasia Hemiparesis	8 days	ChAdOx1 nCoV-19	27	APTT: 24.9	27 546	Positive	Left sigmoid, transverse rectus and inferior longitudinal sinuses thrombosis	None	Fondaparinux and IVIG	None
Castelli	50 M	Headache Vision loss Hemiparesis	11 days	ChAdOx1 nCoV-19	20	NA	>10 000	Negative	Left hemisphere ICH, and lack of opacification of the left transverse and sigmoid sinuses, Uncal herniation	None	Bilateral decompressive craniectomy	Death
De Michele	57 W	Hemiparesis Gaze deviation Dysarthria Neglect	9 days	ChAdOx1 nCoV-19	23	NA	elevated	Positive	Right MCA infarct	None	IV steroids IVIG Decompressive craniectomy	Critical condition
De Michele	55 W	Aphasia Hemiparesis Seizures Coma Abdominal pain	10 days	ChAdOx1 nCoV-19	59	NA	elevated	Positive	Right ICA and bilateral MCA occlusion, Uncal herniation	Portal vein thrombosis, Left lower lobe subsegmental PE	IVIG, and IV steroid	Death

(*Continued*)

**Table 1:** Continued

Author	Age and gender	Presenting symptoms	Duration between vaccination and symptom onset	Administered vaccine	Lowest platelet count (10^9^/L)	Clotting screen on admission	Peak D-Dimer levels	PF4 antibody ELISA	Neuroimaging findings	Extracranial thrombosis	Treatment	Residual neurologic deficits
Bayas	55 W	Chemosis Orbital pain Diplopia Hemiparesis Aphasia Seizure	10 days	ChAdOx1 nCoV-19	30	NA	NA	Positive	Superior ophthalmic vein thrombosis. Ischemic stroke in the MCA territory	None	IV steroids	NA
D’Agostino	54 W	Confusion	12 days	ChAdOx1 nCoV-19	NA	PT: 33.2 APPT: 41	elevated	NA	Right frontal and temporal lobes ICH, Right superior longitudinal sinus, vein of Galen and superior sagittal thrombosis	Floating thrombus within the aortic arch.	Balloon angioplasty of the right coronary artery	Death
Blauenfeldt	60 W	Headache Abdominal pain Confusion	7 days	ChAdOx1 nCoV-19	5	INR: 1 APPT: 28	106 200	Negative	Infarction in the right MCA territory	None	Platelet concentrate	Death
Wolf	22 W	Headache Seizure	4 days	ChAdOx1 nCoV-19	75	NA	2590	Positive	SAH, superior sagittal, left transverse and sigmoid sinuses thrombosis	None	Enoxaparin, followed by dabigatran	None
Wolf	46 F	Headache Aphasia Homonymous hemianopia	8 days	ChAdOx1 nCoV-19	60	NA	22 800	Positive	Superior sagittal, transverse and sigmoid sinuses thrombosis, acute left occipital lobe ICH.	None	Rheolysis via balloon angioplasty, Enoxaparin followed by danaparoid, followed by dabigatran	Mild residual deficits (mRS 1)
Wolf	36 F	Headache Aphasia Confusion	7 days	ChAdOx1 nCoV-19	92	NA	2120	Positive	Thrombosis of the straight and superior sagittal sinuses, Bilateral thalamic edema	None	Danaparoid, followed by Enoxaparin, and dabigatran	None
Mehta	32 M	Headache Hemiparesis Ataxia	9 days	ChAdOx1 nCoV-19	30	NA	NA	NA	Superior sagittal sinus and cortical vein thrombosis and significant cortical oedema with small areas of parenchymal and SAH	None	None	Death
Mehta	25 M	Headache Photophobia Vomiting Rash Gum bleeding Hemiparesis Ataxia	6 days	ChAdOx1 nCoV-19	19	NA	NA	Positive	Superior sagittal sinus thrombosis extending into the cortical veins, SAH and ICH	None	IV unfractionated heparin, platelet transfusions, IV dexamethasone, IVIG	Death
Suresh	27 M	Headaches Vomiting Homonymous hemianopia	2 days	ChAdOx1 nCoV-19	73	PT: 12.9 APTT: 27.5	34 071	Positive	Right transverse venous sinus thrombosis, ICH in the right parietal lobe	None	IVIG Dabigatran Prednisone	Death

(*Continued*)

**Table 1:** Continued

Author	Age and gender	Presenting symptoms	Duration between vaccination and symptom onset	Administered vaccine	Lowest platelet count (10^9^/L)	Clotting screen on admission	Peak D-Dimer levels	PF4 antibody ELISA	Neuroimaging findings	Extracranial thrombosis	Treatment	Residual neurologic deficits
George	40 W	Headache	7 days	Ad26.COV2.S	20	APTT: 26.4	27 150	Negative	Left transverse and sigmoid sinuses thrombosis	Right PE	Bivalirudin IVIG	Recovery with no residual neurologic deficits
Muir	48 W	Malaise Abdominal pain	14 days	Ad26.COV2.S	13	APTT: 41	117 500	Positive	Right transverse and straight sinuses thrombosis	Splanchnic, right hepatic and splenic venous thrombosis	Heparin switched to argatroban and IVIG	Patient remained in critical condition
See	≥40 F	Headaches Hemiparesis	6	Ad26.COV2.S	43	INR: 1.4 APTT: 31	>20	NA	Right transverse, sigmoid and Left IJV thrombosis, Right temporoparietal ICH	None	NA	NA
See	18–39 F	Headaches Aphasia	9	Ad26.COV2.S	78	INR: 1.2 APTT: 22.3	1.1	Positive	Left transverse, sigmoid sinus, confluence of sinuses, and straight sinus thrombosis, Left temporal lobe ICH	None	NA	NA
See	18–39 F	Headaches Hemiparesis Gaze deviation Neglect Seizure	8	Ad26.COV2.S	18	INR: 1.5 APTT: 31.1	8.46	Positive	Superior and inferior sagittal, straight sinuses and cortical venous thrombosis, Bilateral frontal ICH, right SAH and IVH	None	NA	NA
See	18–39 F	Headaches	8	Ad26.COV2.S	127	INR: 1.1 APTT:31.2	5.45	Positive	Right transverse and sigmoid sinuses thrombosis	Portal vein thrombosis and right pulmonary embolus	NA	NA
See	18–39 F	Headaches	6	Ad26.COV2.S	10	INR: 1.1 APTT: 18.1	7.05	Positive	Right transverse and sigmoid sinuses thrombosis	Bilateral lower extremity DVT	NA	NA
See	≥40 F	Headache	13	Ad26.COV2.S	13	INR: 1.2	112.07	Positive	Right transverse and straight sinuses thrombosis, Right IVJ thrombosis, Right occipital ICH	Portal, splenic, right hepatic, distal superior mesenteric venous thrombosis, Right posterior tibial and peroneal DVT	NA	NA
See	18–39 F	Headache Photophobia	15	Ad26.COV2.S	64	INR: 0.9 APTT: 28	7.84	Positive	Superior sagittal, transverse, straight and sigmoid sinuses thrombosis, Right IJV thrombosis	None	NA	NA

(*Continued*)

**Table 1:** Continued

Author	Age and gender	Presenting symptoms	Duration between vaccination and symptom onset	Administered vaccine	Lowest platelet count (10^9^/L)	Clotting screen on admission	Peak D-Dimer levels	PF4 antibody ELISA	Neuroimaging findings	Extracranial thrombosis	Treatment	Residual neurologic deficits
See	18–39 F	Headache	10	Ad26.COV2.S	90	INR: 1.1 APTT: 26.9	6.7	Positive	Right transverse, sigmoid sinuses thrombosis, Right IJV thrombosis	Lower extremity DVT and PE	NA	NA
See	≥40 F	Headache Confusion Hemiparesis Aphasia Seizure	7	Ad26.COV2.S	15	INR: 1.2 APTT: 24.1	>4	Positive	Superior sagittal sinus, bilateral cortical venous thrombosis	None	NA	NA
See	18–39 F	Headaches Photophobia Comma Seizure	7	Ad26.COV2.S	9	INR: 1.2 APTT: 30.2	13.47	Positive	Superior sagittal, right transverse and sigmoid sinuses thrombosis, Right temporal lobe and left cerebellar hemisphere ICH, SAH	None	NA	NA
See	18–39 F	Headaches Neck stiffness Blurry vision	11	Ad26.COV2.S	102	APTT: 26.4	41.71	Positive	Torcula, bilateral transverse, right sigmoid sinuses thrombosis, Bilateral IJV thromboses, Right posterior temporal ICH	None	NA	NA
See	≥40 F	Headaches Neck pain Photophobia	6	Ad26.COV2.S	20		45.57	Positive	Left transverse and sigmoid sinuses thrombosis, Left IJV thromboses	Right PE	NA	NA

Based on these reports, initial recommended investigations included complete blood count, platelet count, coagulation studies (Prothrombin time (PT), INR and PTT), D-dimer, fibrinogen and peripheral blood smear to rule out pseudo-thrombocytopenia and other causes of thrombocytopenia [12]. Other differential diagnoses to evaluate for include: DIC, sepsis, malignancy, thrombotic microangiopathy, systemic lupus erythematosus, antiphospholipid syndrome, paroxysmal nocturnal hemoglobinuria and sickle cell anemia, for which investigations were performed in our case [13]. Similar to the 4Ts score used in HITT evaluation, there has been a proposed 4Ts score for VITT evaluation to improve the diagnostic certainty [14]. The 4Ts score in VITT depends on the degree of thrombocytopenia, the timeline of symptom onset, the history of thrombosis and the presence of alternative diagnosis of thrombosis [15]. Our case had a 4Ts score of 8, which indicated a high probability of VITT.

For treating our patient, we followed the latest guidelines which recommend anticoagulation with direct oral anticoagulants (rivaroxaban, apixaban or dabigatran) or fondaparinux, and treatment with IVIG (1 g/kg/day for 2 days) [6]. Anticoagulation with heparin or warfarin should be avoided [16]. Platelet count should be monitored for recovery, and recovery itself is identified as platelet count of > 150 × 10^9^/mm^3^ [17]. Even in some cases of intracranial hemorrhage, anticoagulation should be considered to prevent progressive thrombosis. Our patient tolerated the treatment with no further complications and achieved complete normalization of his platelet count and neurological symptoms.

There are a few proposed pathophysiologic mechanisms implicated in CVST development. One of these is VITT which is similar to HITT in its pathophysiology. HITT results from the formation of immune complexes consisting of autoantibodies against PF4 and heparin. These immune complexes bind to the surface of platelets and monocytes, provoking their activation by cross-linking Fc *γ*IIA receptors [18]. In the case of VITT, it is believed that the leakage of DNA from the adenovirus infected cells binds to PF4 and triggers the production of autoantibodies [19]. Our case tested positive for PF4 autoantibodies as in previously reported CVST cases associated with Ad26.COV2.S vaccine, which might indicate a high probability of VITT being the underlying pathophysiology of CVST in this patient. Another proposed mechanism of VITT may be independent of PF4 autoantibodies. Viral vector-based COVID-19 vaccines contain high amounts of viral particles, which may be distributed across different body tissues including the brain. The COVID-19 adenoviral vectors might trigger an immune response in the brain, leading to localized vascular thrombosis [20]. According to the reported cases in Table 1, this mechanism could explain CVST in patients who tested negative for PF4 autoantibodies. Interestingly, those patients did not have any associated systemic thrombosis and were reported to have CVST secondary to ChAdOx1 nCoV-19 vaccine.

Despite these recent reports of COVID-19 vaccine-associated CVST, it should be noted that COVID-19 infection itself has been suggested to be a more significant risk factor for CVST. A retrospective study showed that the incidence of CVST after COVID-19 was 39.0 per million (95% CI, 25.2–60.2) compared with any 2-week period in the pre-COVID-19 [21]. There have been a few epidemiologic studies to assess the incidence of CVST during postvaccination period, which has shown conflicting evidence regarding the presence of increased incidence of CVST postvaccination. Epidemiologic studies in Europe regarding the ChAdOx1 nCoV-19 vaccine showed that the incidence of CVST has significantly increased after COVID-19, and greater than what was observed with COVID-19 mRNA vaccines and ChAdOx1 nCoV-19 vaccine [21]. However, when comparing the incidence rate between mRNA-based vaccines and vector-based vaccines, specifically ChAdOx1 nCoV-19 vaccine, the incidence rate was higher in individuals who received ChAdOx1 nCoV-19 [22]. In another epidemiologic study in the United States, there was no increased risk of CVST in the 30 days prior to COVID-19 vaccination (Pfizer-BioNTech, Moderna and Johnson & Johnson) compared with the 30 days after vaccination [23].

## CONCLUSION

Although headaches may be a common side effect of COVID-19 vaccinations, a headache with increased severity should warrant further neuroimaging, and a fundus exam must be performed to assess for intracranial hypertension, a common feature of CVST. Following a thorough workup for hypercoagulability, we were able to intervene in a timely manner, which resulted in an excellent outcome with no residual symptoms or neurological deficits. Our case demonstrates the impact of early recognition of symptoms and signs of CVST in the setting of headache following COVID-19 vaccination.

## Supplementary Material

Supplementary_material_omac154
