# Supplementary material for: Cerebral venous sinus thrombosis after COVID-19 vaccination: a case report and literature review
Source: Oxf Med Case Reports. 2023 Jan 18;2023(1):omac154. doi: 10.1093/omcr/omac154 (PMC9853928; doi:10.1093/omcr/omac154)
Supplement: Supplementary_material_omac154 [file supplementary_material_omac154.docx]

**References of the cases in table 1**

- Graf T, Thiele T, Klingebiel R, Greinacher A, Schabitz WR, Greeve I. Immediate high-dose intravenous immunoglobulins followed by direct thrombin-inhibitor treatment is crucial for survival in Sars-Covid-19-adenoviral vector vaccine-induced immune thrombotic thrombocytopenia VITT with cerebral sinus venous and portal vein thrombosis. J Neurol. 2021;268(12):4483-5.

- Soleimani B, Turaga S, Khan D, Davies C, Duodu Y, Botcherby E, et al. Syndrome of cerebral venous sinus thrombosis and thrombocytopenia after vaccination for COVID-19. Research Square. 2021.

- Zanferrari C, Fanucchi S, Liberato NL, Lauria G, Persico A, Cavallini A. Excellent response to high-dose intravenous immunoglobulin in anti-PF4 positive cerebral thrombosis following Ofxord-AstraZeneca AZD1222 vaccine. Research Square. 2021.

- Castelli GP, Pognani C, Sozzi C, Franchini M, Vivona L. Cerebral venous sinus thrombosis associated with thrombocytopenia post-vaccination for COVID-19. Crit Care. 2021;25(1):137.

- De Michele M, Iacobucci M, Nicolini E, Chistolini A, Pulcinelli F, Cerbelli B, et al. Malignant cerebral infarction, systemic venous thrombosis and thrombocytopenia after ChAdOx1 nCov vaccination: a possible catastrophic variant of vaccine induced thrombotic thrombocytopenia. Research Square. 2021.

- Bayas A, Menacher M, Christ M, Behrens L, Rank A, Naumann M. Bilateral superior ophthalmic vein thrombosis, ischaemic stroke, and immune thrombocytopenia after ChAdOx1 nCoV-19 vaccination. Lancet. 2021;397(10285):e11.

- D'Agostino V, Caranci F, Negro A, Piscitelli V, Tuccillo B, Fasano F, et al. A Rare Case of Cerebral Venous Thrombosis and Disseminated Intravascular Coagulation Temporally Associated to the COVID-19 Vaccine Administration. J Pers Med. 2021;11(4).

- Blauenfeldt RA, Kristensen SR, Ernstsen SL, Kristensen CCH, Simonsen CZ, Hvas AM. Thrombocytopenia with acute ischemic stroke and bleeding in a patient newly vaccinated with an adenoviral vector-based COVID-19 vaccine. J Thromb Haemost. 2021.

- Wolf ME, Luz B, Niehaus L, Bhogal P, Bazner H, Henkes H. Thrombocytopenia and Intracranial Venous Sinus Thrombosis after "COVID-19 Vaccine AstraZeneca" Exposure. J Clin Med. 2021;10(8).

- Mehta PR, Apap Mangion S, Benger M, Stanton BR, Czuprynska J, Arya R, et al. Cerebral venous sinus thrombosis and thrombocytopenia after COVID-19 vaccination - A report of two UK cases. Brain Behav Immun. 2021;95:514-7.

- Suresh P, Petchey W. ChAdOx1 nCOV-19 vaccine-induced immune thrombotic thrombocytopenia and cerebral venous sinus thrombosis (CVST). BMJ Case Rep. 2021;14(6).

- George G, Friedman KD, Curtis BR, Lind SE. Successful treatment of thrombotic thrombocytopenia with cerebral sinus venous thrombosis following Ad26.COV2.S vaccination. Am J Hematol. 2021;96(8):E301-E3.

- Muir KL, Kallam A, Koepsell SA, Gundabolu K. Thrombotic Thrombocytopenia after Ad26.COV2.S Vaccination. N Engl J Med. 2021;384(20):1964-5.

- See I, Su JR, Lale A, Woo EJ, Guh AY, Shimabukuro TT, et al. US Case Reports of Cerebral Venous Sinus Thrombosis With Thrombocytopenia After Ad26.COV2.S Vaccination, March 2 to April 21, 2021. JAMA. 2021.
